# Reproducibility of carotid-femoral pulse wave velocity in end-stage renal disease patients: methodological considerations

**DOI:** 10.1186/s40697-016-0109-6

**Published:** 2016-04-01

**Authors:** Rosendo A. Rodriguez, Valerie Cronin, Timothy Ramsay, Deborah Zimmerman, Marcel Ruzicka, Kevin D. Burns

**Affiliations:** Division of Nephrology, Department of Medicine, The Ottawa Hospital, University of Ottawa, 1967 Riverside Dr., Rm. 535, Ottawa, ON K1H 7W9 Canada; Kidney Research Centre, Ottawa Hospital Research Institute, University of Ottawa, 1967 Riverside Dr., Rm. 535, Ottawa, ON K1H 7W9 Canada; Department of Medicine, The Ottawa Hospital, University of Ottawa, Centre for Practice-Changing Research, Room L-2217, 501 Smyth Road, Ottawa, ON K1H 8L6 Canada; The Ottawa Methods Centre, Ottawa Hospital Research Institute, Centre for Practice-Changing Research, 501 Smyth Road, Ottawa, ON K1H 8L6 Canada

**Keywords:** Aortic stiffness, End-stage kidney disease, Inter-observer variation, Validation studies

## Abstract

**Background:**

In end-stage renal disease (ESRD) patients, increased arterial stiffness detected by carotid-femoral pulse wave velocity (cf-PWV) is associated with fatal cardiovascular events and all-cause mortality. Since cf-PWV is an operator-dependent technique, poor reproducibility may be a source of bias in the estimation of arterial stiffness.

**Objectives:**

We assessed the week-to-week reproducibility of cf-PWV and radial artery pulse wave analysis in healthy subjects and ESRD patients. We also determined the extent of patient eligibility, enrollment, acceptance, and comfort.

**Methods:**

In a cohort study design, independent tonometric examinations of carotid, femoral, and radial arteries were conducted in 20 healthy subjects and 15 ESRD patients attending chronic hemodialysis treatments according to a randomized sequence by two operators on 2 days scheduled 1-week apart. cf-PWV, augmentation index (AIx@HR75) and central pulse pressure (CPP) were the outcome measures. Patients were tested at mid-week and prior to dialysis treatment. The variability on the distance measured between the suprasternal notch and femoral site using two different methods (standard vs direct) was compared. A post-examination survey assessed acceptance and comfort associated with examinations. Reproducibility was evaluated by intra-class correlations (ICCs).

**Results:**

The mean age for healthy subjects and ESRD patients was 45 ± 12 and 63 ± 16 years, respectively. ESRD patients had higher cf-PWV (*p* = 0.0002), elevated AIx@HR75 (*p* = 0.003), and increased CPP (*p* = 0.001) compared to healthy subjects. The mean inter-visit differences for all stiffness indices were non-significant (*p* > 0.05), but the mean inter-operator differences for the cf-PWV were significant only in the healthy subject group (−0.7 m/s; *p* = 0.02). The ICCs between operators and visits were higher for the ESRD group compared to the healthy subjects (between operators, 0.870 vs 0.461; between visits, 0.830 vs 0.570). Distances were longer (*p* < 0.001), but less variable with the standard method compared to the direct method (healthy subjects, *p* = 0.036; ESRD, *p* = 0.39). There was a high rate of patient acceptance and minimal discomfort.

**Conclusions:**

Week-to-week measurements of cf-PWV and pulse wave analysis are highly reproducible in ESRD patients prior to hemodialysis treatment. The high reproducibility and minimal test-to-test variations encourage use of cf-PWV to monitor changes in arterial stiffness and the efficacy of interventions in ESRD patients.

**Trial registration:**

ClinicalTrials.gov, NCT02196610.

**Electronic supplementary material:**

The online version of this article (doi:10.1186/s40697-016-0109-6) contains supplementary material, which is available to authorized users.

## Abrégé

**Mise en contexte:**

L’accroissement de la rigidité des artères présente un risque élevé d’accidents cardiovasculaires ou de mortalité toutes causes confondues chez les patients souffrant d’insuffisance rénale terminale (IRT). La rigidité des artères est décelée par la mesure de la vitesse de l’onde de pouls carotido-fémorale (VOPcf). La technique impliquant l’intervention d’un opérateur, des préoccupations en matière de reproductibilité sont soulevées du fait que celle-ci peut représenter une source de biais dans l’évaluation de cette mesure.

**Objectifs de l’étude:**

L’étude visait à évaluer la reproductibilité semaine après semaine des mesures de la VOPcf et de la VOP au niveau de l’artère radiale chez des sujets sains et des patients atteints d’IRT. On cherchait également à déterminer l’influence de facteurs tels l’admissibilité des patients, leur recrutement ainsi que leur tolérance et leur niveau de confort à l’égard de ces examens.

**Méthodologie:**

Lors de cette étude de cohorte, des examens tonométriques indépendants de la carotide et des artères fémorale et radiale ont été pratiqués sur un groupe de sujets sains (*n* = 20) et un groupe de patients atteints d’IRT (*n* = 15) sous hémodialyse chronique. Les examens ont été réalisés selon une séquence aléatoire, par deux opérateurs différents, à raison de deux séances prévues à une semaine d’intervalle. La VOPcf, l’indice d’augmentation ajusté sur une fréquence cardiaque de 75 bpm (AIx@HR75) et la pression centrale (CPP) ont été employés à titre d’indicateurs de résultats. Les patients ont été testés tout juste avant leur séance d’hémodialyse prévue en milieu de semaine. La variabilité dans la distance mesurée entre l’espace suprasternal et l’emplacement au niveau fémoral a été évaluée en utilisant deux méthodes différentes (méthode régulière et méthode directe) puis comparée. Un sondage remis aux participants à la suite de chaque test a permis de jauger l’acceptation et le niveau d’inconfort lié à l’examen. La reproductibilité du test a été évaluée par coefficient de corrélation inter-classes (ICC).

**Résultats:**

L’âge médian des participants se situait à 45 ± 12 ans pour les sujets sains et à 63 ± 16 chez les patients atteints d’IRT. Ces derniers présentaient des valeurs de VOPcf (*p* = 0,0002) et des AIx@HR75 supérieurs (*p* = 0,003) en plus d’une CCP plus élevée (*p* = 0,001) en comparaison aux valeurs obtenues chez les sujets sains. Dans l’ensemble, aucun des indices de la rigidité des artères n’a démontré de différence significative d’une séance à l’autre (*p* > 0,05) bien qu’une différence significative ait été notée dans la mesure de la VOPcf d’un opérateur à l’autre chez les sujets sains. Les valeurs d’ICC d’un opérateur à l’autre et inter-séance se sont avérées supérieures chez les patients atteints d’IRT lorsque comparées aux valeurs obtenues chez les sujets sains (entre opérateurs : 0,870 vs 0,461; inter-séances : 0,830 vs 0,570). Les distances mesurées entre l’espace suprasternal et l’emplacement fémoral se sont révélées plus longues (*p* < 0,001), mais moins variables lorsqu’évaluées par la méthode normalisée que par la méthode directe (sujets sains : *p* = 0,036; patients en IRT: *p* = 0,39). Finalement, les sondages post-examens ont indiqué un taux élevé d’acceptation et un inconfort minimal pour les participants.

**Résultats:**

Il apparait que les mesures de la VOPcf et l’analyse de l’onde de pouls sont très reproductibles d’une semaine à l’autre chez les patients atteints d’IRT lorsque mesurées tout juste avant la séance d’hémodialyse. Forte d’une reproductibilité élevée et doublée d’une variabilité minimale d’essai en essai, la mesure de la VOPcf s’avère une technique recommandée pour suivre les changements dans la rigidité artérielle chez les patients atteints d’IRT et assurer l’efficacité des interventions auprès de cette clientèle.

## What was known before

Carotid-femoral pulse wave velocity has been used to assess cardiovascular risk in studies involving patients with chronic kidney disease. Only few studies have assessed the reproducibility of this technique.

## What this adds

Week-to-week measurements of the carotid-femoral pulse wave velocity, augmentation index, and central pulse pressures performed by two operators are highly reproducible with minimal test-to-test variations in end-stage renal disease (ESRD) patients prior to hemodialysis treatment. High concordance between operators can be achieved if training is undertaken, preferably on at least 20 subjects before any clinical study.

## Introduction

Arterial stiffness is an age-related physiologic process that is associated with a decreased ability of conductive arteries to absorb pulse pressure [1]. Age-adjusted increase in arterial stiffness represents an important risk factor for cardiovascular disease in the general population [2]. In patients with chronic kidney disease (CKD), increased arterial stiffness may be one of the earliest detectable manifestations of adverse structural and functional changes within the vessel wall [3]. Moreover, the premature aging of the vascular system that follows the progression from CKD to ESRD leads to extreme increases in arterial stiffness [4], a phenomenon that has been associated with adverse cardiovascular events [5–8]. Accordingly, instruments that reliably measure arterial stiffness can be valuable tools in the assessment of cardiovascular risk and can be used to monitor the efficacy of therapeutic strategies in CKD patients.

Since the aorta is the principal capacitive element in the arterial tree, non-invasive measurements of stiffness in the aorta by the carotid-femoral pulse wave velocity (cf-PWV) reflect the physiologic effects of elevated arterial stiffness on the heart [1, 6]. Indeed, cf-PWV is an excellent predictor of fatal cardiovascular events and all-cause mortality in ESRD patients [7, 9, 10]. Because the cf-PWV is an operator-dependent technique [11–13], poor reproducibility could represent an important source of bias in the estimation of clinically significant changes in arterial stiffness for ESRD patients. An acceptable level of concordance between operators, however, would reassure the use of this technique as a predictor of cardiovascular risk [11, 12]. As a prelude to adopting measures of arterial stiffness in ESRD patients at our center to assess cardiovascular risk, we set out to determine the reproducibility of measuring the cf-PWV and radial artery pulse wave analysis (PWA) between two operators at two different times in healthy subjects and ESRD patients receiving hemodialysis treatments. We also determined the extent of patient eligibility and enrollment at our center and the level of acceptance and comfort associated with these non-invasive vascular assessments.

## Methods

The study was approved by the Ottawa Health Science Network Research Ethics Board (Protocol # 20140457) and registered at ClinicalTrials.gov (NCT02196610.)

### Study population

#### Healthy subjects group

Healthy staff from the Division of Nephrology and Kidney Research Centre at the Ottawa Hospital Research Institute and The Ottawa Hospital were invited to participate in the study. Health status was defined by a self-reporting questionnaire obtained by telephone interview, prior to enrollment. Subjects were considered suitable for enrollment if they were >18 years in age, had no history of hypertension, diabetes mellitus, cancer, or cardiovascular, kidney, liver, or neurologic disease, and were not taking prescription medications. Current pregnancy was an exclusion criterion, and subjects with body mass index ≥30 and active smoking within the past 6 months were also excluded. Two non-invasive measurements of arterial blood pressure (BP) with an automated Omron device (Omron Healthcare Inc; Hoofddorp, Netherlands) prior to testing completed the health assessment. Subjects were eligible if the diastolic BP was ≤90 mm Hg and systolic BP ≤140 mm Hg. In all subjects who met the healthy enrollment criteria, written informed consent for participation was obtained.

#### ESRD group

All adult patients (>18 years) with stage 5 CKD who attended chronic hemodialysis treatments at The Ottawa Hospital, Riverside Campus for at least 3 months were screened for eligibility. Exclusion criteria included a history of atrial fibrillation, active cancer or history of cancer in the past 5 years, pregnancy, neuromuscular conditions that limited ambulatory ability, implanted mechanical, bioprosthetic heart valves or pacemakers, inability to measure BP in at least one arm, or pre-dialysis systolic BP ≥200 mm Hg within the previous six hemodialysis treatments. Patients whose smoking habit was >15 cigarettes per day in the last 6 months were also excluded. Written informed consent was obtained in all patients who met eligibility requirements and agreed to participate.

#### Study protocol

Examinations were conducted at the same time of the day on 2 days scheduled 1 week apart (visits 1 and 2). For healthy participants, no consumption of alcohol was allowed for 24 h before the examination, and no tea, coffee, or smoking for at least 4 h before the examination. ESRD patients followed the same preparation rules as healthy subjects except for those with diabetes mellitus who had a light meal before examination. Measurements in ESRD patients were conducted 1 h before the start of the hemodialysis treatment, at the mid-week hemodialysis session. For both healthy subjects and ESRD patients, the brachial BP was recorded as the average of at least two of three BP recordings measured by an automated Omron BP device. In ESRD patients, demographic data were collected from the patients’ medical records at enrollment including age, sex, history of diabetes mellitus, hypertension, dyslipidemia, cardiovascular history, and medications.

All hemodynamic assessments including cf-PWV were independently performed by two operators at visit 1 (baseline) and visit 2 (follow-up) with an applanation tonometry device (Millar, SPT-301 B, Houston, TX, USA) and the SphygmoCor^®^ hardware and software (version 9.1, AtCor Medical, Sydney, Australia). The order of testing by the two operators was randomly assigned using computer-generated random numbers. Randomization allocation was concealed using opaque envelopes that were opened prior to testing. Operators were blinded to the final interpretation of the arterial stiffness indices. Healthy subjects were tested first in the study. Once the majority of healthy subjects were tested (i.e., 80 %), the assessment of ESRD patients commenced. The technique for cf-PWV has been described elsewhere [1, 3, 11–13]. In brief, we identified the site of the strongest pulse in the carotid and femoral sites and the pulse pressure waves were scanned using a pencil-like tonometry device and transferred to the SphygmoCor apparatus, which calculated the mean recorded pulse waveform (10 s), obtained sequentially at both arteries. A three-lead electrocardiogram (ECG) determined the time difference between the ECG-“R” wave and the foot of the pulse pressure wave, so the transit time of the pulse wave between these two arterial sites was calculated. The operators independently estimated the traveling distance of the pulse wave by performing surface tape measurements between the suprasternal notch-to-the carotid site (proximal distance) and between the suprasternal notch-to-umbilicus-to-femoral site (distal distance) [1]. The final aortic path length was calculated by subtracting the distance between the suprasternal notch and the carotid site from the sum of the distance between the suprasternal notch and umbilicus plus umbilicus and femoral site. Measurements of the carotid-femoral distance were repeated at follow-up and the new values entered in the calculations. Since the distance is critical for calculating the cf-PWV, we compared the variation of our standard method of distal length against a straight measurement of the distance between the suprasternal notch and the femoral site [14]. In patients with unilateral arteriovenous fistulas, tonometric measurements were performed on the side contralateral to the fistula. To control for the quality of recorded waveforms, we accepted the best of two recordings if the pulse waves had adequate height (≥80 mV), height variations less than 15 %, and standard deviation of the time difference between the ECG-“R” wave and the pulse wave signals from the two sites ≤10 % [11].

#### PWA

Pulse wave velocity in the radial artery was recorded to derive the central blood pressure profile, using a validated transfer algorithm from the SphygmoCor^®^ software [15]. Similar to the cf-PWV examination, we controlled for the quality of the recorded waveforms by accepting the best of two recordings if the following criteria were met: adequate height (>80 mV), height variations less than 15 %, and an operator index ≥75 % obtained from the SphygmoCor device [11]. Using the central blood pressure profiles, the following parameters were calculated: (a) the central pulse pressure (CPP) as the difference between the estimated systolic and diastolic central blood pressure and (b) the augmentation index with correction for a heart rate of 75 beats per minute (AIx@HR75) as the difference between the first and the second systolic peaks (P1, P2) divided by the central pulse pressure.

#### Patient acceptance and comfort

After the second visit, healthy subjects and ESRD patients were asked to complete a questionnaire that included five closed-ended questions on a 5-point Likert-style scale that assessed their level of acceptance and comfort associated with the examinations (Additional file 1).

#### Statistical analyses

Values are provided as means ± SD, unless otherwise stated. Bland-Altman plots were used to assess the variability in the measurements between the two operators and the 2 week-to-week visits for the index values of cf-PWV, CPP, and AIx@HR75. The variability and reproducibility of each parameter was expressed in terms of mean differences between paired measurements and their 95 % confidence intervals (CI). Histograms, residual plots and the Kolmogorov-Smirnov statistic were used to assess the normal distribution of continuous variables. Rates and proportions between the two groups were evaluated by Fischer or *X*^2^ tests and the significance of the differences between groups or visits by Student *t* tests. The inter-operator reproducibility and the reliability between visits were assessed by intra-class correlations (ICCs) and 95 % CI. A *p* value <0.05 was used to indicate statistical significance (two-sided). Analyses were performed using SPSS version 23.0 (IBM Co; Armonk, NY, USA).

## Results

### Study populations

**Fig. 1:**
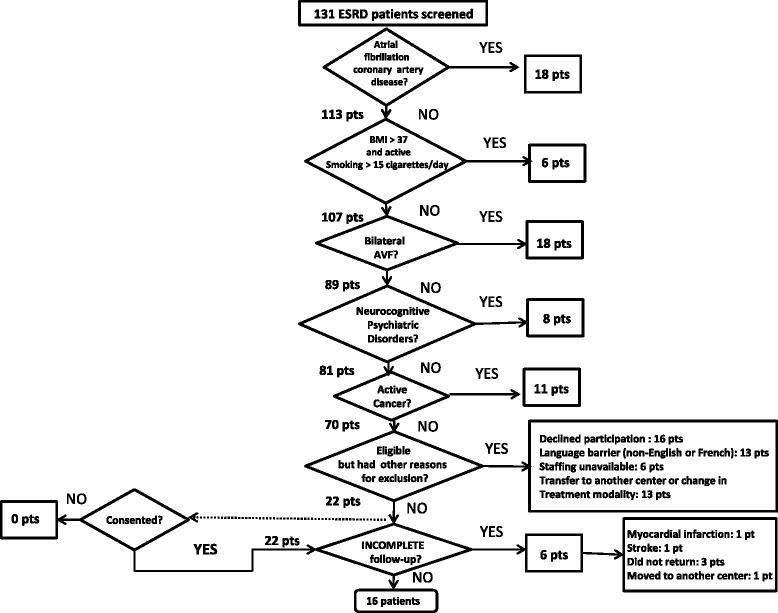
Flow chart of screening and enrollment procedure in end-stage renal disease patients. Fifty-three percent (70/131) of the original screened patients remained eligible; 16 of the 22 patients who consented completed the full study. Atrial fibrillation and presence of bilateral arteriovenous fistulas accounted for 59 % of exclusions. *AVF* arteriovenous fistula, *BMI* body mass index, *ESRD* end-stage renal disease

For healthy subjects, 20 of 45 screened subjects met enrollment criteria and all consented to participate in the study. Exclusions were related to medical conditions (*n* = 22), body mass index (BMI) >32 (*n* = 2) and active smoking (*n* = 1). A total of 131 ESRD patients on hemodialysis were screened at our center. Figure 1 illustrates the proportions of screened, eligible, and consenting ESRD patients with the most frequent reasons for exclusion. Seventy of the 131 screened ESRD patients were deemed eligible (53 %), but only 22 consented to the testing procedure (31 %). All 20 participants in the healthy subjects group completed both baseline and follow-up examinations, but only 16 of the 22 patients in the ESRD group completed all examinations.

Table 1 shows the demographic data for participants in the two groups. Patients in the ESRD group were significantly older (*p* < 0.001) than healthy subjects, but there was no difference in gender distribution, weight, height, or rate of smoking (<6 months) between the two groups (*p* > 0.10). In the ESRD group, the prevalence of diabetes mellitus, hypertension, arteriovenous fistula (AVF), cardiovascular disease, and previous renal transplantation was 38, 75, 75, 50, and 13 % respectively. In the ESRD group, obesity in one patient limited examination of the femoral artery while previous surgical resection of the radial artery in the contra-lateral AVF arm of another patient precluded PWA. Thus, the final analyses for each stiffness modality included 15 patients.

**Table 1:** Demographic characteristics of healthy subjects and ESRD patients

Parameter	Healthy subjects group (%)	ESRD group (%)
Number of subjects	20	16
Males	12/20 (60)	10/16 (63)
Paired measurements (baseline and follow-up)	20/20 (100)	15/16 (94)
Age (years)	45 ± 12	63 ± 16***
Weight (kg)	77 ± 10	78 ± 21
Height (m)	1.74 ± 0.10	1.70 ± 0.10
SBP (mmHg)	118 ± 12	142 ± 24**
DBP (mmHg)	71 ± 9	67 ± 11
Smoking (last 6 months)	0 (0)	0 (0)
Diabetes	0 (0)	6/16 (38)
Hypertension	0 (0)	12/16 (75)
Cardiovascular disease (coronary artery disease, heart failure, stroke, TIA, PVD)	0 (0)	8/16 (50)
AVF	0 (0)	12/16 (75)
Previous renal transplant	0 (0)	2/16 (13)
Anti-hypertensive medication	0 (0)	12/16 (75)

*AVF* arteriovenous fistula, *DBP* diastolic blood pressure, *ESRD* end-stage renal disease, *MI* myocardial infarction, *PVD* peripheral vascular disease, *SBP* systolic blood pressure, *TIA* transient ischemic attack

**p < 0.01; ***p < 0.001

### Between-group differences in arterial stiffness

All carotid and femoral artery waveforms were within accepted quality standards. ESRD patients had significantly higher cf-PWV (visit 1, *p* = 0.0002; visit 2, *p* = 0.0006), elevated augmentation indices (visit 1, *p* = 0.003; visit 2, *p* = 0.037) and increased central pulse pressures (visits 1 and 2, *p* = 0.001) compared to healthy subjects. Tables 2 and 3 display the mean operator and mean visit values for the cf-PWV, AIx@HR75, and CPP obtained by the two operators at the two visits. The mean (±SD) cf-PWV in the ESRD group were 8.6 ± 2.4 and 8.8 ± 3.6 m/s at visits 1 and 2, compared to 6.6 ± 1.9 and 6.2 ± 1.2 m/s, respectively, in the healthy subjects group. The mean AIx@75HR was 24 % ± 14 and 23 % ± 11 for the two visits in ESRD patients and 14 % ± 15 and 17 % ± 14 in the healthy subjects group. Finally, the central pulse pressures achieved mean values of 58 ± 21 and 56 ± 21 mmHg in the ESRD group compared to 35 ± 5 and 35 ± 6 mmHg in the healthy subjects group.

**Table 2:** Mean operator values and mean inter-operator differences for the carotid-femoral pulse wave velocity measurements and radial artery pulse wave analysis

Parameter	Group	Mean operator values	Mean difference	*p* value
		±SD	(95 % CI)	
Carotid-femoral PWV				
cf-PWV (m/s)	HS	6.3 ± 1.2	−0.7 (−1.3 to −0.11)	0.020
	ESRD	8.7 ± 2.9	−0.4 (−1.2 to 0.4)	0.390
Subtracted cf-distance (mm)	HS	466 ± 33	+7.9 (2.3 to 13.5)	0.007
	ESRD	470 ± 36	+8.4 (0.1 to 16.8)	0.050
cf mean time (ms)	HS	77 ± 12	+8.2 (3.1 to 13.2)	0.002
	ESRD	60 ± 21	+5.7 (0.4 to 11.0)	0.040
SBP (mmHg)	HS	117 ± 12	−2.3 (−5.3 to 0.7)	0.130
	ESRD	138 ± 22	+1.3 (−3.3 to 5.9)	0.580
DBP (mm Hg)	HS	70 ± 9	−1.3 (−2.9 to 0.3)	0.100
	ESRD	67 ± 10	+0.9 (−1.2 to 2.9)	0.620
Radial artery PWA				
AIx@HR75 (%)	HS	15 ± 14	−0.5 (−2.7 to 1.7)	0.630
	ESRD	24 ± 11	−1.5 (−6.2 to 3.1)	0.520
Central pulse pressure (mm Hg)	HS	35 ± 5	+0.3 (−1.3 to 1.9)	0.680
	ESRD	57 ± 20	+0.3 (−3.0 to 3.5)	0.870

*AIx@HR75* augmentation index corrected for heart rate at 75 beats per minute, *cf* carotid-femoral, *CI* confidence interval, *DBP* diastolic blood pressure, *ESRD* end-stage renal disease, *HS* healthy subjects, *PWA* pulse wave analysis, *PWV* pulse wave velocity (m/s), *SBP* systolic blood pressure

**Table 3:** Mean visit values and mean differences between visits (baseline and follow-up) as measured by the same operators in the healthy subjects and ESRD groups

Parameter	Group	Mean visit values	Mean difference	*p* value
		± SD	(95 % CI)	
Carotid-femoral PWV				
cf-PWV (m/s)	HS	6.4 ± 1.3	−0.4 (−1.0 to 0.1)	0.130
	ESRD	8.7 ± 2.8	+0.1 (−0.75 to 1.0)	0.780
Subtracted cf-distance (mm)	HS	465 ± 31	+1.0 (−7.3 to 9.2)	0.820
	ESRD	470 ± 35	−6.9 (−18.8 to 5.0)	0.240
cf mean time (ms)	HS	77 ± 13	+0.6 (−3.7 to 4.9)	0.780
	ESRD	60 ± 21	+1.7 (−4.3 to 7.6)	0.571
SBP (mmHg)	HS	117 ± 12	−2.3 (−5.3 to 0.7)	0.130
	ESRD	137 ± 19	−6.9 (−14.2 to 0.5)	0.070
DBP (mmHg)	HS	70 ± 9	−1.3 (−2.9 to 0.3)	0.100
	ESRD	67 ± 10	−2.6 (−5.3 to 0.2)	0.070
Radial artery PWA				
AIx@HR75 (%)	HS	15 ± 14	+2.6 (−0.1 to 5.2)	0.053
	ESRD	23 ± 11	−7.7 (−3.8 to 2.2)	0.610
Central pulse pressure (mm Hg)	HS	35 ± 5	−0.4 (−2.2 to 1.5)	0.680
	ESRD	57 ± 20	−2.0 (−7.0 to 3.1)	0.430

*AIx@HR75* augmentation index corrected for heart rate at 75 beats per minute, *cf* carotid femoral, *CI* confidence interval, *DBP* diastolic blood pressure, *ESRD* end-stage renal disease, *HS* healthy subjects, *PWA* pulse wave analysis, *PWV* pulse wave velocity (m/s), *SBP* systolic blood pressure

### Variability in cf-PWV measurements

Table 2 summarizes the mean inter-operator differences and Table 3 the mean inter-visit differences in cf-PWV, subtracted carotid-femoral distances, carotid-femoral mean times, and systolic and diastolic BPs for both groups. Table 4 summarizes the ICC coefficients and 95 % CIs for stiffness indices. The between-operator differences for cf-PWV and carotid-femoral distances were significant in the healthy subjects group, but not in the ESRD group (Table 2). The mean inter-operator differences for the baseline and follow-up assessments were −0.5 m/s (95 % CI, −1.2 to 0.23) and −0.6 m/s (95 % CI, −1.3 to −0.04), respectively. Moreover, the between-operator differences in the recorded carotid-femoral mean times were significantly different in both groups. No significant differences were identified in the systolic and diastolic BP recordings for both groups. In the healthy subjects group, analyses of the Bland-Altman plots showed that operator 1 consistently overestimated the cf-PWV relative to operator 2 by a mean difference of 0.7 m/s. This difference in the cf-PWV measurements was associated with operator 1 consistently recording smaller carotid-femoral distances and shorter carotid-femoral mean times compared to operator 2 (see Table 2). This was in contrast to measures in the ESRD group, where the mean cf-PWV differences between the two operators were smaller (−0.4 m/s). Thus, the level of concordance was higher for the ESRD group compared to the healthy subjects group (ICC, 0.870 vs 0.461).

**Table 4:** Intra-class correlation (ICC) coefficients for the level of agreement between operators and visits (baseline and follow-up) in the healthy subjects and end-stage renal disease groups

Parameter	Groups	Inter-operator	95 % CI	Between-visits	95 % CI
cf-PWV	HS	0.461	0.2 to 0.7	0.570	0.20 to 0.77
	ESRD	0.87	0.72 to 0.94	0.830	0.64 to 0.92
AIx@HR75	HS	0.942	0.89 to 0.97	0.914	0.84 to 0.95
	ESRD	0.660	0.28 to 0.83	0.864	0.71 to 0.94
CPP	HS	0.780	0.6 to 0.88	0.680	0.39 to 0.83
	ESRD	0.950	0.90 to 0.98	0.883	0.75 to 0.94

*AIx@HR75* augmentation index corrected for heart rate at 75 beats per minute, *cf* carotid femoral, *CI* confidence interval, *CPP* central pulse pressure, *ESRD* end-stage renal disease, *HS* healthy subjects

The mean differences between the two visits for cf-PWV, subtracted carotid-femoral distances, carotid-femoral mean times, and systolic and diastolic BP were non-significant (Table 3). The ICCs for the cf-PWV between the two visits was higher in the ESRD group compared to that for the healthy subjects group (ICC, 0.83 vs 0.57). The mean cf-PWV differences between baseline and follow-up were +0.1 m/s in the ESRD group and −0.4 m/s for the healthy subjects group.

To confirm that enhanced training of the operators had an effect on the magnitude of the inter-operator differences, all participants were organized in small blocks of 10, 10, and 15 participants according to the chronological order in which they were tested. After testing the first 10 participants, the inter-operator difference for the cf-PWV was −1.03 m/s (95 % CI, −2.070 to 0.008; *p* = 0.05), but this difference decreased to −0.35 m/s (95 % CI, −0.94 to 0.25; *p* = 0.242) after the second group of 10 participants was completed. This variability was comparable to the mean inter-operator difference of −0.41 m/s (95 % CI, −1.19 to 0.37; *p* = 0.288) achieved after finishing the last 15 participants.

### Variability in PWA measurements

All radial artery pulse wave recordings were within quality standards, and the mean operator quality indices were 90 % ± 10 and 89 % ± 7 at baseline and 90 % ± 10 and 91 % ± 8 at follow-up for both operators. All mean differences between operators and visits were non-significant in both groups (Tables 2 and 3). The mean inter-operator differences for the AIx@HR75 at baseline and follow-up were −2.0 % (95 % CI, −6.0 to 2.0) and −0.4 % (95 % CI, −3.0 to 3.0), respectively. For the CPP, the mean differences were 0.0 mm Hg (95 % CI, −2.0 to 1.9) and 0.5 mmHg (95 % CI, −2.3 to 3.4), respectively. The level of concordance between operators and visits for the AIx@HR75 was consistently higher in the healthy subjects group (ICC, 0.942 and 0.914) compared to the ESRD group (ICC, 0.660 and 0.864). For the CPP, however, a higher level of agreement was observed in the ESRD group (ICC, 0.950 and 0.883) relative to the healthy subjects group (ICC, 0.780 and 0.680).

### Differences in distance measurements

A comparison between the two methods of distance measurement was performed in 10 subjects in the healthy subjects group and 12 patients in the ESRD group. The distal distance was longer with the standard method compared to the direct method. The average difference between the two methods was 14.9 mm ± 12 (580 mm vs 565 mm; *p* < 0.001) in the healthy subjects group and 12.2 mm ± 10 (595 mm vs 582 mm) in the ESRD group (*p* < 0.001). Most importantly, the mean differences between operators were significantly larger with the direct method compared to the standard method in both groups. In the healthy subjects group, the mean inter-operator difference was 14.4 mm ± 21 with the direct method, but only 4.2 ± 25 mm with the standard method (*p* = 0.036). In the ESRD group, these differences decreased to −5.3 ± 17 mm with the direct method and −2.5 ± 14 mm with the standard method (*p* = 0.39).

### Participant acceptance and comfort

Nineteen healthy subjects and 16 ESRD patients completed our acceptance and comfort survey. The mean duration of testing by the 2 operators was 62 ± 8 min and only 1 of 16 patients (6 %) perceived that the procedure was too long. Most ESRD patients (15/16) and all healthy subjects (19/19) reported no discomfort. All participants (100 %) considered that both testing procedures (cf-PWV and PWA) were not harmful and that the information provided to them in terms of preparation and testing was complete. Moreover, the majority of patients (15/16) and healthy subjects (18/19) agreed to participate in studies involving repeated measurements of arterial stiffness.

## Discussion

Increased arterial stiffness as determined by measurements of cf-PWV is an independent marker of cardiovascular risk in the general population and a major contributor to mortality in ESRD patients [2, 3, 6, 16]. Since measurement of cf-PWV is an operator-dependent technique, cases of poor reproducibility and significant day-to-day variation may become important sources of bias in the estimation of changes in arterial stiffness. This issue may be particularly important for ESRD patients who may be already subject to significant variations in cf-PWV related to comorbidities and BP variability due to fluctuations in extracellular fluid volume. Thus, an acceptable level of concordance between operators and week-to-week examinations would reassure the use of this technique to predict cardiovascular risk in any clinical study. We found that cf-PWV measurements in ESRD patients are highly reproducible when examinations are performed on a weekly basis by two independent operators. Nevertheless, our results showed large inter-operator differences in the cf-PWV for the healthy subjects group that were not found in the ESRD group (see Table 2). As testing of the healthy subjects group was completed first, it is likely that the higher level of concordance between operators in the ESRD group simply reflects enhanced training at the time of the examination. Our operators, who had no previous experience with use of the SphygmoCor device, received theoretical and hands-on training for 4 weeks prior to study initiation. Our results suggest, however, that this training period may not be sufficient and that testing of at least 20 healthy subjects would be necessary to bring the discrepancy between the two operators to a small difference and to achieve an acceptable inter-operator agreement in ESRD patients.

Our mean inter-operator differences in the ESRD group were comparable to the mean differences between operators in the study by Fridmodt-Møller et al. [11] in 19 CKD patients (stages 3–5) using the same tonometric device (0.4 vs 0.3 m/s), but our week-to-week variations were smaller than their day-to-day variations (0.1 vs −0.7 m/s). These differences, however, should be considered in the context of the significance for the cardiovascular risk of ESRD patients. An increase of 1 m/s in ESRD patients increases both cardiovascular and overall mortality by 34 % using crude estimates and 14 % after adjusted analyses [7]. In our study, the technical error linked to weekly assessments is minimal and represents only 1.4 % of the estimated risk predicted by cf-PWV [7]. Our data support use of these devices for serial examinations since cf-PWV values remain stable when tests are repeated by the same operator within a short period of time (i.e., 1 week).

The level of agreement between operators and the week-to-week concordance of the AIx@HR75 and CPP were good to excellent in both groups and slightly better than the cf-PWV. The higher reproducibility of these two stiffness indices might have been related to better detection of radial artery pulses compared to carotid and femoral arteries. Interestingly, concordance between operators for the central pulse pressure was higher in the ESRD group than that in the healthy subjects group. As ESRD patients tended to have wider pulse pressures than healthy participants, this may have permitted improved identification of radial artery pulse pressures at the time of tonometry scanning.

A clinical study [17] has shown that an increase of 10 % in the augmentation index in ESRD patients is associated with an increase in cardiovascular mortality by 48 % (HR, 1.48; 95 % CI, 1.16−1.90) and in all-cause death by 51 % (HR, 1.51; 95 % CI, 1.23–1.86). In addition, an increase of 23 mmHg (1 SD) in the CPP augments the risk for all-cause death by 20 % (HR, 1.2; 95 % CI, 0.9–1.5) [5]. In considering our inter-operator variability in the performance of the technique, our technical error associated with the AIx@HR75 and CPP represent only 7.2 and 0.2 %, respectively, of the overall risk predicted by these two arterial stiffness parameters in ESRD patients.

An important methodological consideration when measuring cf-PWV is the degree of variability in the measurement of carotid-femoral distance. Although our standard method resulted in longer distances compared to the direct measurement, the latter is subject to larger inter-operator variability compared to the standard method. Previous studies have shown that differences in the method of distance measurement can lead to differences in cf-PWV by up to 30 % [17–21]. Moreover, as distance measurements depend on the anthropometric features of participants, this may affect the relationship between cf-PWV and cardiovascular risk [14]. For tall and/or obese individuals, cf-PWV is less likely to achieve the same predictive value, compared to shorter and lean individuals [14]; an important consideration during the design of clinical studies since approximately 60 % of dialysis patients are overweight or obese at the time of kidney transplantation [22]. Consequently, the method of distance measurement, standardization of the chosen method and proper assessment of the operator-variability are important factors to consider prior to initiation of a clinical study. To minimize variability associated with repeated measures, the use of a single measured distance for all subsequent examinations should be considered. We recommend that this methodological information be explicitly included in publications to improve reliability between studies.

The cf-PWV values in our healthy subjects group were within the range of the lower 2 standard deviations from the age-adjusted mean values obtained by the “Arterial Stiffness’ collaboration” group [23]. Several methodological differences between the two studies deserve mention. First, we used the subtraction method for the carotid-femoral distance and these values were not converted to an equivalent of direct total distance. Second, we used the intersecting tangent algorithm in the SphygmoCor to calculate cf-PWV, although use of different algorithms from a variety of devices may account for differences in calculations from 5 to 15 % [23, 24]. Third, due to our small sample size, a more precise relationship between cf-PWV and age was not estimated. In this regard, previous studies indicate that cf-PWV increases at a rate of 0.1 m/s per year with more pronounced increases above age 55 years [12, 25].

A major concern in any clinical study is the effectiveness of patient recruitment and retention. Almost half of the chronic hemodialysis population at one hospital campus was eligible for this study, but only one in three patients consented to participate. We excluded patients with conditions that may affect the recording of cf-PWV such as atrial fibrillation, bilateral arteriovenous fistulas, severe coronary artery disease, obesity, and active smoking (Fig. 1). These comorbidities accounted for most excluded patients. In addition, 6 (27 %) of the 22 ESRD patients who had initially consented were lost to follow-up. In two patients, this was due to medical complications (i.e., stroke and myocardial infarction) and four patients did not return for the second assessment. More convenient scheduling times for assessments, tools to engage patients, and delivery of positive reinforcement may assist with patient recruitment for such non-invasive studies. Finally, patient and subject self-assessment of this procedure showed a high level of acceptance and tolerability for these measurements and for repeated testing.

### What does this study add?

Although the reproducibility of measuring cf-PWV has been previously assessed in ESRD patients, our study represents an improvement on previous studies by the addition of a randomization sequence in the order of testing, concealment of the allocation order, and blinding of the final interpretation of the arterial stiffness indices by the two operators. Although it is reasonable to expect that higher experience and increased level of training would improve the level of inter-operator agreement, this assumption has always been supported on empirical bases. We believe that our study provides reasonable support for the minimal level of training necessary to achieve an acceptable level of concordance between the operators. Despite the fact that measurements of the distance from the sternal notch-to-umbilicus-to-femoral site are recommended for the calculation of the cf-PWV in the SphygmoCor^®^ device, the operator variability on these measurements is unclear. Our study provides information on the inter-operator variability and compares this method of distance measurement with a more direct measurement between the sternal notch and femoral site.

## Conclusions

Our study found that the measurement of cf-PWV and PWA by applanation tonometry is a highly reproducible test in ESRD patients examined at mid-week immediately prior to hemodialysis treatment. Week-to-week variations in measures were minimal and level of patient comfort and acceptability of the technique was satisfactory. Two important sources of variation in cf-PWV are the method for distance measurement and the level of operator training. The standard distance from the suprasternal notch-to-umbilicus-to-femoral site resulted in larger distances but less variability as compared with the direct distance from suprasternal notch-to-femoral site. To achieve an acceptable level of concordance between operators, training should be undertaken, preferably on at least 20 subjects before any clinical study. Despite some minor differences between operators, arterial stiffness indices remain stable if tests are repeated within a short period of time. The high reproducibility and minimal test-to-test variations found in our study encourage the use of this technique to monitor changes in arterial stiffness and the efficacy of interventions in ESRD patients.

## Additional file

Additional file 1: Participant Acceptance and Comfort Survey. Five-point Likert-style questionnaire for assessing the acceptance and comfort to the testing procedure of arterial stiffness in Healthy Subjects and End-Stage Renal Disease patients. The survey was delivered to participants at the end of the second applanation tonometry examination (follow-up). (DOCX 29 kb)
