# Supplementary material for: Reproducibility of carotid-femoral pulse wave velocity in end-stage renal disease patients: methodological considerations
Source: Can J Kidney Health Dis. 2016 Apr 1;3:20. doi: 10.1186/s40697-016-0109-6 (PMC4818522; doi:10.1186/s40697-016-0109-6)
Supplement: Additional file 1: — Participant Acceptance and Comfort Survey. Five-point Likert-style questionnaire for assessing the acceptance and comfort to the testing procedure of arterial stiffness in Healthy Subjects and End-Stage Renal Disease patients. The survey was delivered to participants at the end of the second applanation tonometry examination (follow-up). (DOCX 29 kb) [file 40697_2016_109_MOESM1_ESM.docx]

**Additional file 1**

**Please check the response that best describes your feelings with regards to the testing procedure.**

**1**. **The duration of the testing procedure was too long.**

| I strongly disagree    ○ ○ | I disagree  ○ | neither agree or disagree  ○ | I agree  ○ | I strongly agree  ○ |
| --- | --- | --- | --- | --- |
| **2. I experienced discomfort during the arterial stiffness testing.** | | | | |
| I strongly disagree | I disagree | neither agree or disagree | I agree | I strongly agree |
| ○ | ○ | ○ | ○ | ○ |
| **3. This test was harmful to me.** | | | | |
| I strongly disagree | I disagree | neither agree or disagree | I agree | I strongly agree |
| ○ | ○ | ○ | ○ | ○ |
| **4. The information provided by the research coordinator about this test was clear to me.** | | | | |
| I strongly disagree | I disagree | neither agree or disagree | I agree | I strongly agree |
| ○ | ○ | ○ | ○ | ○ |

**5. I would agree to have this test repeated every 6 months, if it was available to me.**

I strongly disagree I disagree neither agree or disagree I agree I strongly agree

○ ○ ○ ○ ○
